# Competition between biodetoxification fungus and lactic acid bacterium in the biorefinery processing chain for production of cellulosic L-lactic acid

**DOI:** 10.1186/s40643-024-00772-6

**Published:** 2024-05-23

**Authors:** Zhibin Li, Lingxiao Zhang, Niling He, Bin Zhang, Jie Bao

**Affiliations:** State Key Laboratory of Bioreactor Engineering, East China University of Science and Technology, 130 Meilong Road, Shanghai, 200237 China

**Keywords:** Lignocellulose, Biodetoxification, High temperature, L-lactic acid fermentation, *Paecilomyces Variotii* FN89, *Pediococcus acidilactici* ZY271

## Abstract

Biodetoxification fungus selectively degrades toxic inhibitors generated from pretreatment of lignocellulose without consuming fermentable sugars. However, one barrier for practical application is the sustained cell viability in the consequent fermentation step to compete the fermentable sugars with fermenting strains, resulting in sugar loss and reduced target product yield. This study investigated the competitive growth property between the biodetoxification fungus *Paecilomyces variotii* FN89 and the L-lactic acid bacterium *Pediococcus acidilactici* ZY271 under varying temperature and lactic acid osmatic stress. The results show that the L-lactic acid bacterium *Ped. acidilactici* ZY271 showed less thermotolerance to *Pae. variotii* FN89 at high temperature of 45 °C to 50 °C in both synthetic medium and wheat straw hydrolysate. In the higher temperature environment, the growth of the biodetoxification strian failed to compete with the lactic acid fermentation strain and was quickly eliminated from the fermentation system. The high temperature fermentation facilitated a fast transition from the detoxification stage to the fermentation stage for higher production of L-lactic acid.

**Supplementary Information:**

The online version contains supplementary material available at 10.1186/s40643-024-00772-6.

## Introduction

L-lactic acid fermentation from lignocellulose feedstock provides a sustainable supply of chiral lactic acid monomer for polylactic acid (PLA) production (He et al. 2022; Reshmy et al. 2022). Lignocellulose biorefinery chain includes the steps of pretreatment, hydrolysis, detoxification, fermentation, and purification, in which the pretreatment step generates considerable furan aldehydes, weak organic acids, and phenolic compounds as toxic inhibitors for the consequent fermentation strains (Klinke et al. 2004; Yang et al. 2018). Among the various detoxification methods, biodetoxification shows the most promising option by the complete and selective degradation of the inhibitors without consuming fermentable sugars (He et al. 2022; Yi et al. 2019; Zhang et al. 2010, 2021a).

One challenge for practical biodetoxification applications in biorefinery processing chain is the microbial growth competition between the biodetoxification strains and the fermentation strains when the biodetoxification step is transferred to the fermentation step. The high priority and selectivity of the biodetoxification strains on the inhibitors are only valid before the complete removal of inhibitors (Yi et al. 2019). After the inhibitors are almost removed, the biodetoxification strains start to consume the fermentable sugars and result in an ineffective consumption of the fermentable sugars (Zhang et al. 2021b). Therefore, a fast transition from detoxification to fermentation is crucially important and needed to avoid sugar loss by curbing the viability of biodetoxification strains and increasing the viability of lactic acid fermentation strains in the fermentation step.

Direct autoclave inactivation is not feasible to achieve this fast transition because the cellulase enzymes are deactivated by autoclave operation resulting in the failure of the following simultaneous saccharification and co-fermentation (SSCF) (Liu et al. 2018). Antifungal agents are also not preferred because the agents also affect the viability of the fermentation strains and create problems in the downstream purification of target products (Marchese et al. 2016). Here, we proposed a high temperature (45–50 °C) fermentation strategy to achieve the fast transition from biodetoxification to fermentation by directly inoculating the lactic acid bacterium *Pediococcus acidilactici* ZY271 into the freshly biodetoxified lignocellulose hydrolysates containing the biodetoxification fungus *Paecilomyces variotii* FN89. The engineered *Ped. acidilactici* ZY271 strain efficiently converts the fermentable sugars derived from lignocellulose into high optical purity (99.6%) L-lactic acid at relatively high temperature (42 °C) (Qiu et al. 2018). The high temperature stress, along with the high L-lactic acid titer stress, quickly and effectively suppressed the cell viability of fungus *Pae. variotii* FN89. During the high temperature fermentation, the thermal tolerant L-lactic acid bacterium *Ped. acidilactici* ZY271 showed higher viable competitiveness than the fungus *Pae. variotii* FN89. Meanwhile, the rates of sugar consumption and L-lactic acid generation were also significantly improved. This strategy achieved the fast transition from detoxification to fermentation in biorefinery processing and could be applied in general biorefinery fermentations using thermal stable strains.

## Materials and methods

### Strains

The engineered lactic acid bacterium *Pediococcus acidilactici* ZY271 (CGMCC #13,611) was utilized for L-lactic acid fermentation, which was cultured as fermentation seed anaerobically at 42 °C under mild stirring (150 rpm) in simplified man-rogosa-sharp (MRS) medium contained 20 g/L glucose, 10 g/L peptone, 10 g/L yeast extract, 5 g/L sodium acetate, 2 g/L ammonium citrate dibasic, 2 g/L dipotassium phosphate, 0.58 g/L magnesium sulfate heptahydrate, 0.25 g/L manganese sulfate monohydrate (Qiu et al. 2018). 1% (v/v) of the glucoamylase GA-L-NEW enzyme (Genencor, Jilin, China) was used to avoid cell flocculation in seed culture (Liu et al. 2015).

*Paecilomyces variotii* FN89 (CGMCC #17,665) is a biodetoxification fungus and selectively degrades most inhibitors in pretreated wheat straw (Zhang et al. 2010). The strain was incubated on potato dextrose agar (PDA) medium and cultured at 37 °C for the massive accumulation of spores. Then the spores were collected and inoculated into synthetic medium described in our previous study (Zhang et al. 2021b) at 10% (v/v) inoculum size, 37 °C, and 300 rpm. The biodetoxification seed culture was obtained after 20 h.

### Reagents

Commercial cellulase enzyme Cellic CTec 2.0 was purchased from Novozymes China (Beijing, China), with 256 FPU/mL of filter paper activity, 4653.3 CBU/mL of cellobiase activity, and 86.3 mg/mL of protein content according to the method from National Renewable Energy Laboratory (NREL) (Adney and Baker 1996), Ghose (Ghose 1987), and Bradford (Bradford 1976) respectively. Glucose, xylose, and other reagents were of analytical grade and obtained from Titan Scientific Co. (Shanghai, China). Yeast extract and peptone were purchased from Oxoid (Hampshire, UK).

### Wheat straw feedstock and biorefinery operations

Wheat straw was purchased from Jining, Shandong, China, in Spring, 2021, then coarsely chopped, washed, air-dried, and crushed through a mesh of 10 mm in diameter. There was 37.5% of cellulose, 22.7% of xylan, 25.4% of lignin, and 8.89% of ash in the wheat straw by the NREL protocols (Sluiter et al. 2008, 2012).

The dry acid pretreatment of wheat straw was performed in a 20 L reactor according to our previous protocols (He et al. 2014; Zhang et al. 2011). The pretreated wheat straw solids were neutralized to about pH 5.0 by adding 20% (w/w) Ca(OH)_2_ slurry and mixing evenly. Then the pre-hydrolysis of pretreated feedstock was conducted at 30% (w/w) of solids loading, 50 °C, 150 rpm for 12 h in a 5 L bioreactor. The added cellulase dosage was 5 mg cellulase proteins or 14.8 FPU per gram of dry matter.

Then the pre-hydrolyzed slurry was biodetoxified by inoculating the fungus *Pae. variotii* FN89 to selectively degrade the inhibitors (Zhang et al. 2021a). Briefly, 10% (v/v) of biodetoxification seed culture (~ 3 × 10^8^ spores) was inoculated into the pretreated wheat straw slurry and cultured at 37 °C, 750 rpm, and 1 vvm without pH regulation (Zhang et al. 2021b). The biodetoxification was completed (16–20 h) when the pH value reached a peak value and started to decline. The pretreated wheat straw contained 15.4 mg acetic acid, 1.6 mg 5-hydroxymethylfurfural (HMF), and 1.3 mg furfural per gram of dry matter before biodetoxification. After the biodetoxification, these inhibitors were completely removed and no inhibitors were detected by HPLC.

### Lactic acid fermentation

The competitive cell growth was investigated by lactic acid fermentation in simplified MRS medium. Briefly, the *Ped. acidilactici* ZY271 and *Pae. variotii* FN89 seeds cultures were co-inoculated at 10% (v/v) inoculum size of each strain in a 1 L bioreactor containing 600 mL simplified MRS medium with 60 g/L glucose and 40 g/L xylose (Zhao et al. 2013). The fermentation lasted for 48 h at different temperatures (37 °C, 45 °C, and 50 °C), 150 rpm. The pH was controlled at 5.5 by adding 25% (w/w) calcium hydroxide slurry.

The cellulosic lactic acid fermentation was conducted in wheat straw hydrolysate by SSCF. The biodetoxified wheat straw hydrolysates (containing the *Pae. variotii* FN89 cells without inactivated treatment) were directly inoculated with *Ped. acidilactici* ZY271 seeds at 10% (v/v) size and the nutrient solution (He et al. 2022) to start SSCF. The SSCF was conducted at different temperatures (37 °C, 45 °C, and 50 °C), 300 rpm, and pH 5.5. Samples were collected from the fermentation broth every 24 h. All the mentioned fermentations were conducted in duplicate.

### Analysis methods

The cell viability was roughly evaluated by observing the growth trend of colony forming unit (CFU) on the MRS medium agar plates (Penfornis et al. 2016). Briefly, 100 µL of 10^− 2^ diluted broth sample was dropped and stretched on MRS medium agar plates, then cultured at 42 °C for 24 ~ 48 h to form colonies. The lactic acid yield in SSCF was calculated by the method mentioned in our previous study (He et al. 2022).

Glucose, xylose, L-lactic acid, furfural, HMF, and acetic acid were analyzed by the previously described HPLC method (Qiu et al. 2018).

## Results and discussion

### Competitive cell growth of biodetoxification fungus and lactic acid bacterium

In biodetoxification stage, the inhibitory compounds (furfural, HMF, acetic acid, and most phenolic aldehydes) are selectively and completely removed by the biodetoxification fungus strain *Pae. variotii* FN89, and the remaining phenolic aldehydes (4-hydroxybenzaldehyde, vanillin, and syringaldehyde) existed at a minor level and have a limited inhibitory effect on *Ped. acidilactici* ZY271 (Qiu et al. 2020). When the biodetoxification transits to the L-lactic acid fermentation stage, the challenge is the microbial growth competition between the remaining fungus *Pae. variotii* FN89 with high cell viability and the inoculated bacterium *Ped. acidilactici* ZY271 as fermentation seeds. To achieve a fast transition from detoxification to fermentation and avoid sugar losses by the fungus, the cell viability of *Pae. variotii* FN89 should be efficiently curbed and the viability of *Ped. acidilactici* ZY271 should be maintained high. A gap of culture temperature exists between *Pae. variotii* FN89 (favored 37 °C (Zhang et al. 2021a) and *Ped. acidilactici* ZY271 (favored 45 °C for fermentation and 42 °C for seed culture (Zhao et al. 2013). Three temperatures were selected to assay the cell viability: 37 °C, the most favorable temperature for the growth of the biodetoxification strain *Pae. variotii* FN89; 45 °C, the most favorable temperature for the growth of the fermentation strain *Ped. acidilactici* ZY271; 50 °C, the optimal temperature for cellulase enzyme for enzymatic hydrolysis during the simultaneous saccharification and lactic acid co-fermentation.

**Fig. 1:**
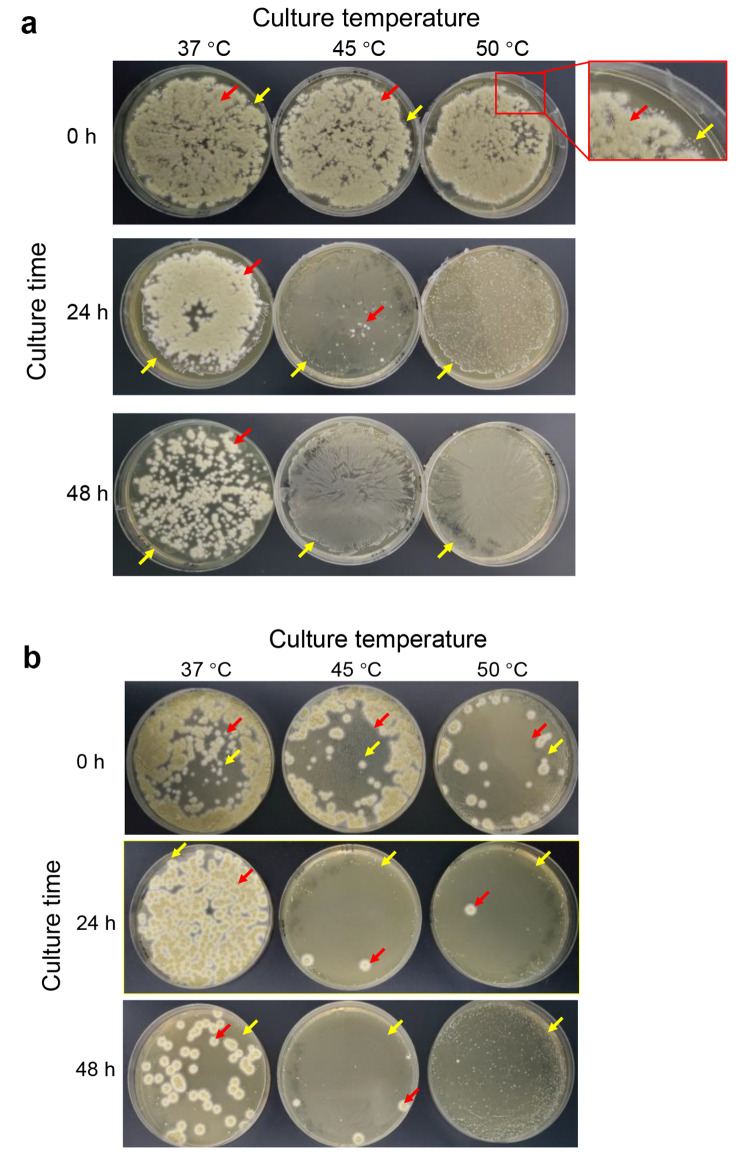
(**a**) Co-cultured *Pae. variotii* FN89 and *Ped. acidilactici* ZY271 in synthetic MRS medium (**b**) Co-cultured *Pae. variotii* FN89 and *Ped. acidilactici* ZY271 in wheat straw hydrolysate Cell viability of the co-cultured *Pae. variotii* FN89 and *Ped. acidilactici* ZY271 in synthetic MRS medium (a) and in wheat straw hydrolysate (b). In (a), the co-culture was conducted at 150 rpm, pH 5.5, and 10% (v/v) inoculum size in 1 L bioreactor containing 600 mL of simplified MRS medium. In (b), the co-culture was performed in 3 L reactor with 1 L of the pretreated and biodetoxified wheat straw slurry at 300 rpm, pH 5.5, and 10% (v/v) inoculum size. 100 µL of 10^− 2^ diluted samples were stretched on agar plates and cultured for 24 h at 42 °C. The red arrows indicate the colonies of *Pae. variotii* FN89 and the yellow arrows indicate the bacterial colonies or lawns of *Ped. acidilactici* ZY271

The competitive cell growth was firstly conducted in both the synthetic MRS medium and wheat straw hydrolysate generally by co-inoculation and co-culture at 37 °C, 45 °C, and 50 °C, respectively (Fig. 1). Two different kinds of colonies (big ones, *Pae. variotii* FN89; small ones, *Ped. acidilactici* ZY271) grown on the agar plates of both medium agar plates. The colonies of *Pae. variotii* FN89 were big and flocculent (the red arrows), consisting of mycelia and spores, while the colonies of *Ped. acidilactici* ZY271 appeared milky white lawns or small and round colonies (the yellow arrows). The fungus grows much faster and its colonies were much bigger than the lactic acid bacteria and the lactic acid bacterium colonies were overlapped by the biodetoxification fungus colonies, thus the accurate CFU counting of the two co-cultured microbes were practically not possible. Therefore, the cell viability of the two strains was roughly measured by the observation of the growing trends on agar plates.

Figure 1(**a**) showed that at the preferred temperature for fungus growth (37 °C), *Pae. variotii* FN89 grew vigorously during the competitive cell growth as indicated by the dense colonies on the MRS agar plates, while the growth of *Ped. acidilactici* ZY271 was less competitive with small colonies covered by the fungus. At 45 °C, the viability of *Pae. variotii* FN89 cells decreased sharply with only a few colonies after 24 h, while the thermal tolerant *Ped. acidilactici* ZY271 showed vigorous growth and formed a dense cell colony lawn. At 50 °C, almost no fungus colony of *Pae. variotii* FN89 appeared and the bacterium colonies of *Ped. acidilactici* ZY271 formed the dense lawns. Similar to the cell growth in synthetic medium, Fig. 1(**b**) showed that *Pae. variotii* FN89 grew vigorously with more colonies than *Ped. acidilactici* ZY271 wheat straw hydrolysate at 37 °C, and was sharply suppressed at 45 °C and 50 °C with few colonies, while *Ped. acidilactici* ZY271 dominated the cell growth and formed dense colony layers. The result indicates the cell viability of the biodetoxification fungus *Pae. variotii* FN89 was quickly suppressed at higher temperatures suitable for *Ped. acidilactici* ZY271. In another word, the lactic acid-producing strain won the cell growth competition and dominated the co-culture cell community during the high temperature fermentation stage.

**Fig. 2:**
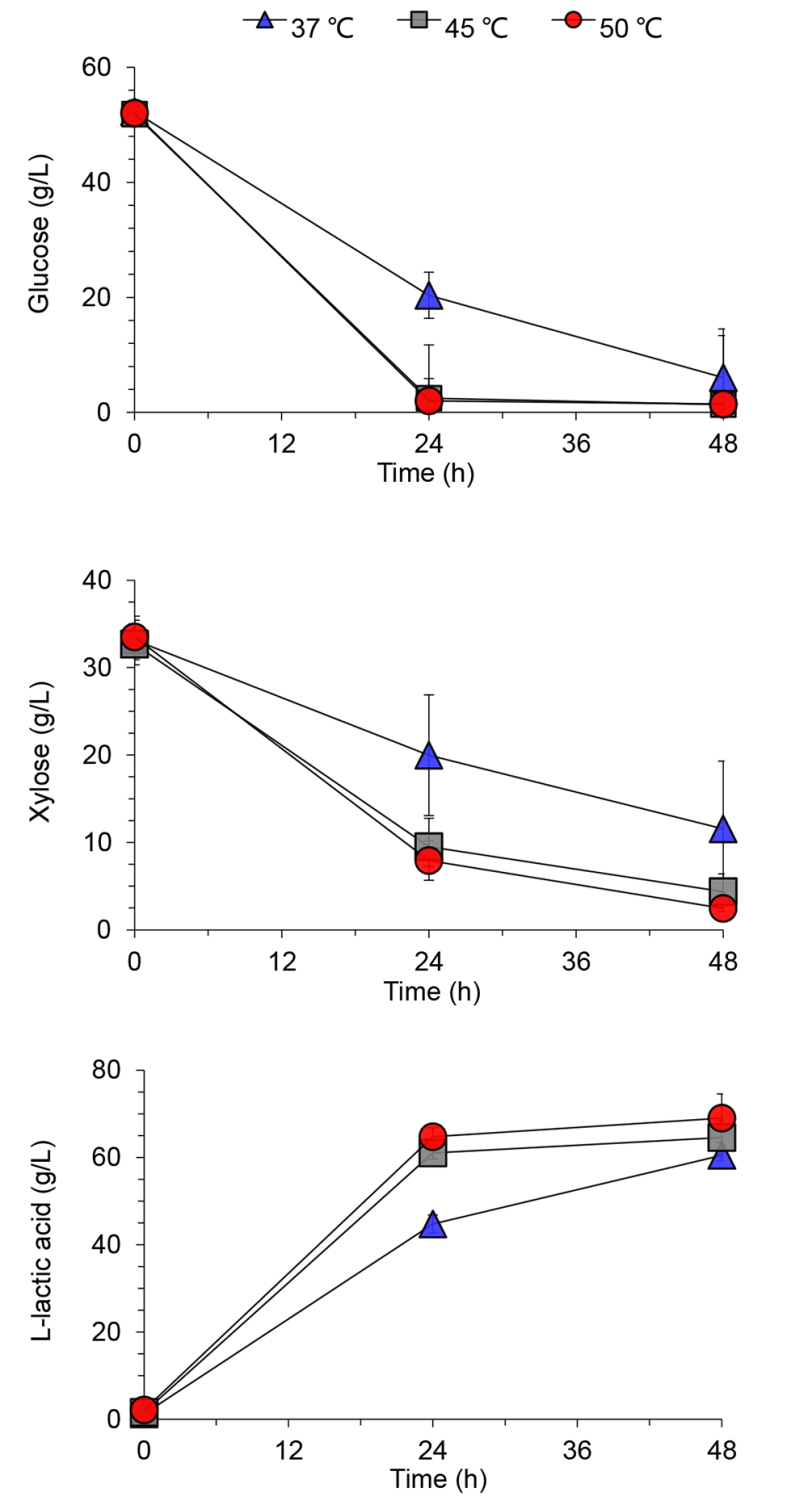
Sugars consumption and L-lactic acid generation during co-culture of *Ped. acidilactici* ZY271 and *Pae. variotii* FN89 in simplified MRS medium. The conditions are shown in the figure captions of Fig. 1(**a**)

Figure 2 showed that the utilization rates of glucose were improved by 61% and the xylose utilization was doubled at both 45 °C and 50 °C than that at 37 °C in the competitive cell growth, while the L-lactic acid generation improved by 14% during the first 24 h and by 10% at the end, owing to the strong competition capacity of the lactic acid bacterium *Ped. acidilactici* ZY271 to the biodetoxification strain *Pae. variotii* FN89 at high fermentation temperatures. The co-culture at 50 °C also showed a preferable lactic acid yield of 0.96 g/g sugar and productivity of 1.44 g/L/h, compared with that of 0.83 g/g sugar and 1.26 g/L/h at 37 °C.

### Osmatic stress of high-titer L-lactic acid on biodetoxification fungus *Pae. Variotii* FN89

Lactic acid generally generates osmatic stress on microbial growth and thus influences cell viability (Batish et al. 1997; Chen et al. 2021; León Peláez et al. 2012). *Ped. acidilactici* ZY271 produced cellulosic L-lactic acid up to the maximum of ~ 130 g/L (Qiu et al. 2018). Therefore, the accumulated lactic acid during the co-culture may cause high osmatic stress on the biodetoxification fungus and suppress its viability.

**Fig. 3:**
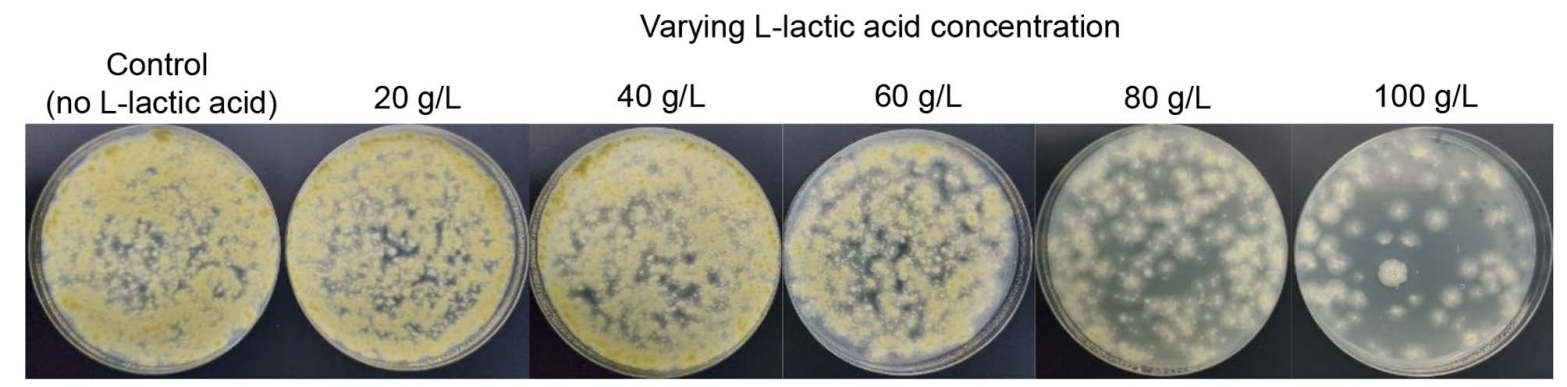
Cell viability of *Pae. variotii* FN89 under varying L-lactic acid concentrations (0–100 g/L) in the wheat straw hydrolysate. The fermentation was conducted in 250 mL shake flasks containing 50 mL of wheat straw hydrolysate at 37 °C, 300 rpm, pH 5.5, and 10% (v/v) inoculum size. The sampling was conducted after 24 h, and 100 µL of 10^− 2^ diluted fermentation broth was stretched on PDA medium agar plates and cultured at 37 °C for 24 h

**Fig. 4:**
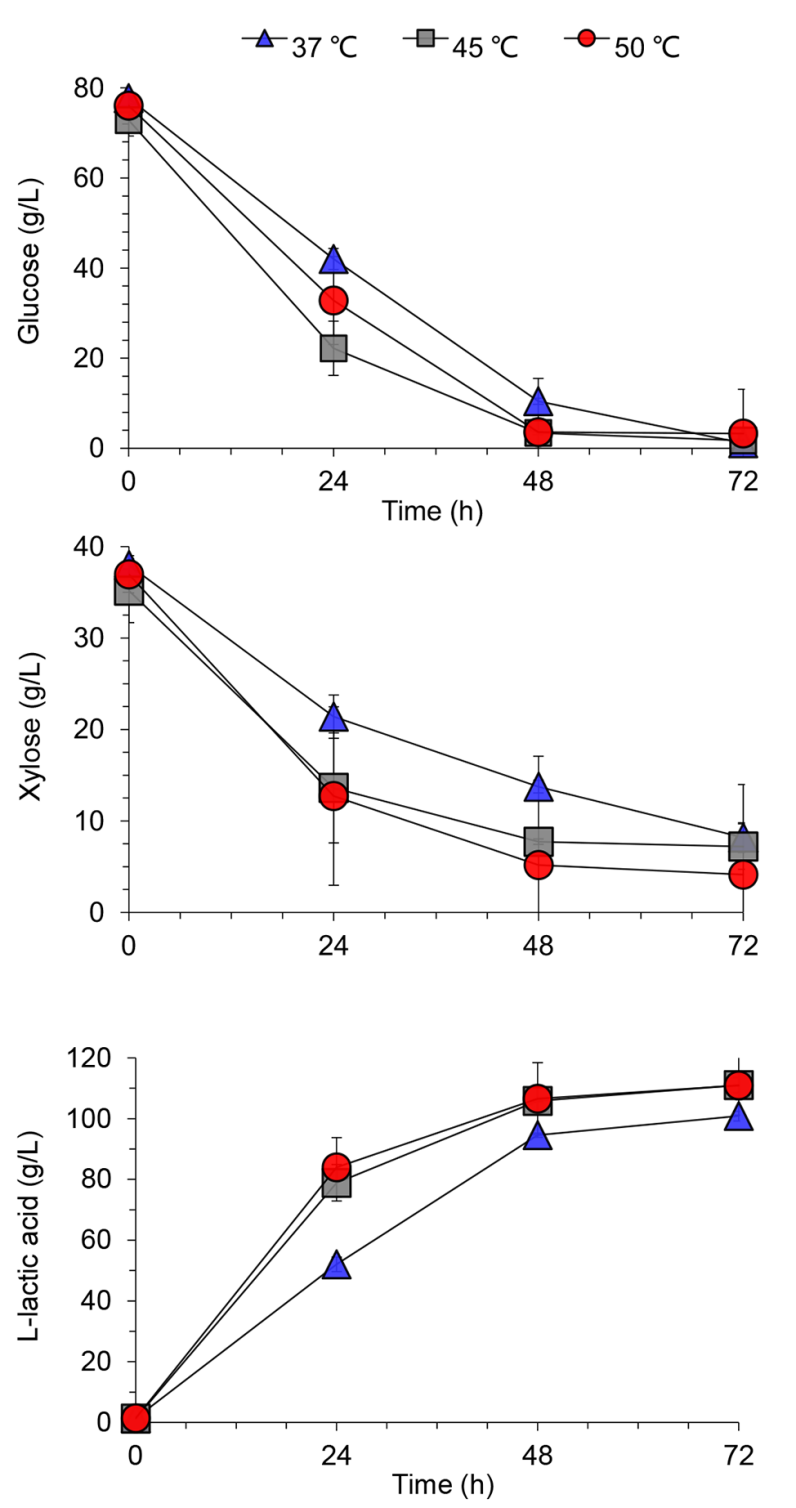
Competitive cellulosic L-lactic acid fermentation by SSCF in wheat straw hydrolysates. The SSCF was carried out in the biodetoxified wheat straw hydrolysates containing the cells of *Pae. variotii* FN89, at 37 °C, 45 °C, and 50 °C, respectively, 300 rpm, pH 5.5, and 10% (v/v) inoculum size

The osmatic stress of high-titer L-lactic acid on fungus *Pae. variotii* FN89 was investigated by culturing it in wheat straw hydrolysate under the L-lactic acid range of 0 ~ 100 g/L (Fig. 3). When at below 40 g/L of L-lactic acid, *Pae. variotii* FN89 exhibited vigorous growth with the dense colonies on the agar plates. When the lactic acid concentration reached 60 ~ 100 g/L, the colony density gradually decreased and the colony scattered at 100 g/L of L-lactic acid. This trend shows the cell viability of *Pae. variotii* FN89 was suppressed by a higher L-lactic acid concentration of 100 g/L. Considering the final L-lactic acid concentration was close to ~ 130 g/L in the optimal cellulosic L-lactic acid fermentation, the osmatic suppression on the biodetoxification fungus existed and played a role in suppressing the viability of *Pae. variotii* FN89 cells along with the high temperature. Although the suppression of high lactic acid concentration was not as strong as the high temperature (Figs. 1 and 3, and 4), the high rate of lactic acid generation under high temperatures let this kind of suppression come earlier.

### Cellulosic L-lactic acid fermentation after transition from biodetoxification to fermentation

The cellulosic L-lactic acid fermentation was conducted in the form of SSCF using wheat straw after the fast transition from biodetoxification to fermentation by high temperatures (45 °C and 50 °C).

The final lactic acid titer at 37 °C was 10% smaller than that at 45 °C and 50 °C with similar glucose and xylose consumption conversion, suggesting an additional 10% sugar loss at 37 °C (Fig. 4). The lactic acid yield and productivity reached 0.87 g/g and 1.54 g/L/h at 45 °C and 50 °C, respectively, compared with 0.78 g/g and 1.40 g/L/h at 37 °C. This lower lactic acid yield and productivity may be due to the fierce competition of fungus *Pae. variotii* FN89 to the lactic acid bacterium *Ped. acidilactici* ZY271 at the lower temperature, and a portion of the fermentable sugars were utilized by the fungus. While the viability of fungus *Pae. variotii* FN89 cells were suppressed at 45 °C and 50 °C, so the sugar loss caused by the fungus was also avoided. On the other hand, the lactic acid bacterium *Ped. acidilactici* ZY271 reached its optimal temperature at 45 ~ 50 °C and showed a better fermentation performance with faster sugar consumption and lactic acid generation. The higher cellulase activity at higher temperature and the osmatic stress by lactic acid on *Pae. variotii* FN89 may also play a role during SSCF for lactic acid production. Therefore, the fast transition from biodetoxification to fermentation was achieved mainly by conducting the high-temperature fermentation using a thermal stable L-lactic acid bacterium *Ped. acidilactici* ZY271, together with the high cellulase activity in the biorefinery process and the osmatic stress of high-titer lactic acid. This strategy effectively and easily solves the problem of the inactivation of biodetoxification fungus by using thermal tolerant fermentation strains.

In biorefinery chain of lignocellulose conversion, inhibitor generation from pretreatment step makes the major difference with the general industrial fermentation using starch or sucrose as carbon feedstocks. The harsh inhibition of furfural, 5-hydroxymethylfurfural (HMF), acetic acid, and phenolic compounds on fermentation strains is a lethal factor in the cell viability and metabolism performance for the production of biofuels (such as ethanol) and biochemicals (such as lactic acid) from lignocellulose feedstock (Yi et al. 2019; Zhang et al. 2010). Various inhibitor removal methods have been tried and biodetoxification has been proven to be the best option for its extensive inhibitor degradation, wastewater-free, very low sugar loss, and easy operation behaviors (Guo et al. 2022). However, one major concern comes into light when biodetoxification is practically applied to biorefinery conversion: the competing property of biodetoxification strain with fermentation strains. When its mission of biodetoxification to degrade the inhibitors is fulfilled and the process turns to the fermentation step for production of biofuels or biochemicals, the growing competition between biodetoxification strains and fermentation strains starts. Generally, biodetoxification strains are highly tolerant to environmental stress and also generally survive at least a certain period of fermentation step. The survival of biodetoxification strain in fermentation step will consume a considerable amount of fermentable sugars and curb the growth and metabolism of fermentation strains, resulting in low yield and low productivity of fermentation products (Zhang et al. 2021b).

In this study, we demonstrated a perfect case for overcoming this barrier by L-lactic acid fermentation from wheat straw through dry biorefining. The high level of inhibitors from dry acid pretreatment of wheat straw was completely removed by a unique biodetoxification fungus *Pae. variotii* FN89, then the biodetoxified wheat straw was fermented into L-lactic acid by an engineered thermal-tolerant lactic acid bacterium *Ped. acidilactici* ZY271. The results showed that the high temperature of up to 50 °C and the high titer of L-lactic acid product played key roles in curbing the growth (and consequently autolyzed) of biodetoxification fungus *Pae. variotii* FN89 and elevating the growth and metabolism of the fermentation bacterium *Ped. acidilactici* ZY271. The anaerobic condition without oxygen supply was also one of the reasons for curbing the growth of biodetoxification fungus. The high cellulosic L-lactic acid production was observed from wheat straw feedstock.

This high temperature fermentation strategy achieves the fast transition between biodetoxification and high-performance fermentation. It could be applied in biorefinery fermentation using different thermal tolerance or thermophilic strains. Lactic acid bacteria such as *Bacillus coagulans* (Zhang et al. 2014) and *Enterococcus faecium* (Abdel-Rahman et al. 2014) with temperature tolerance up to 50 °C have the potential to achieve the fast transition in lignocellulose biorefinery fermentations. This method also suggests that the fast transition between biodetoxification and fermentation could be reached by constructing process conditions (such as high temperature, high osmatic stress from product accumulation, and anaerobic conditions), which are favorable for fermentation strains and unfavorable for biodetoxification when biodetoxification step is transferred into fermentation step. This principle provides an important scenario for designing the optimal biorefinery process for the production of various biofuels and biochemicals.

## Conclusion

High-temperature fermentation, along with the fast accumulation of high-titer lactic acid, achieved the fast transition from detoxification to fermentation in biorefinery production of cellulosic L-lactic acid. The high-thermal tolerance strain *Ped. acidilactici* ZY271 had an overwhelming cell growth advantage over the biodetoxification fungus *Pae. variotii* FN89 at high temperatures, showing satisfying fermentation performance with significant improvement in sugar consumption and L-lactic acid generation rates. This study provides a feasible strategy of high temperature fermentation to suppress the viability of the detoxification fungus without any extra steps to closely link these two indispensable parts in the lignocellulose biorefinery chain.

### Electronic supplementary material

Below is the link to the electronic supplementary material.

Supplementary Material 1

## Data Availability

The data will be available on the reasonable request.
