# Supplementary figures and images for: Competition between biodetoxification fungus and lactic acid bacterium in the biorefinery processing chain for production of cellulosic L-lactic acid
Source: Bioresour Bioprocess. 2024 May 23;11(1):54. doi: 10.1186/s40643-024-00772-6 (PMC11116323; doi:10.1186/s40643-024-00772-6)

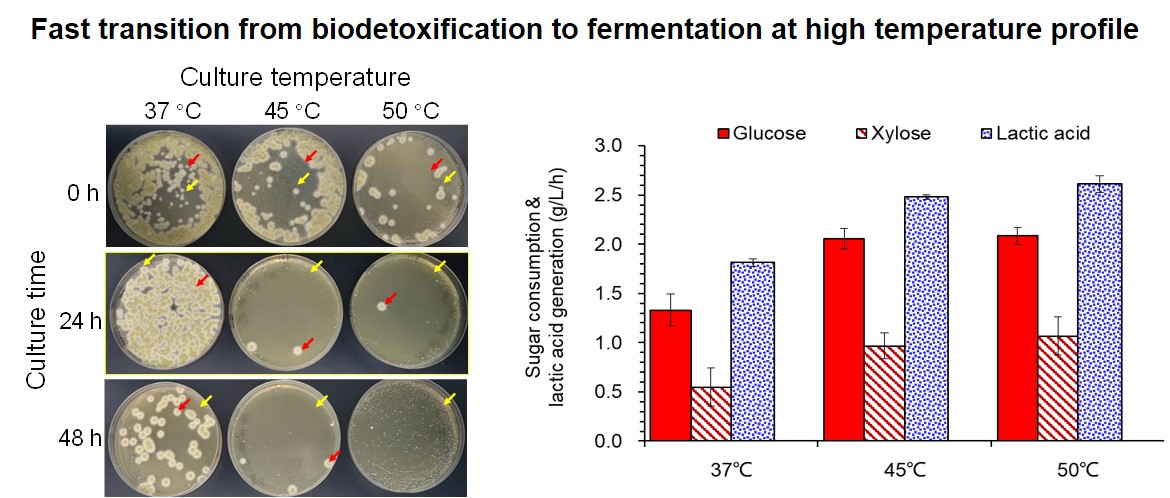

Supplement: Supplementary file 1 — Supplementary Material 1 [file 40643_2024_772_MOESM1_ESM.jpg]
